# COVID-19 global pandemic planning: Performance and electret charge of N95 respirators after recommended decontamination methods

**DOI:** 10.1177/1535370220976386

**Published:** 2020-12-16

**Authors:** Anne M Grillet, Martin B Nemer, Steven Storch, Andres L Sanchez, Edward S Piekos, Jonathan Leonard, Ivy Hurwitz, Douglas J Perkins

**Affiliations:** 1 Thermal/Fluid Component Sciences, Sandia National Laboratories, Albuquerque, NM 87185, USA; 2 Diagnostic Science and Engineering, Sandia National Laboratories, Albuquerque, NM 87185, USA; 3 WMD Threats and Aerosol Science, Sandia National Laboratories, Albuquerque, NM 87185, USA; 4 Department of Internal Medicine, Center for Global Health, University of New Mexico Health Science Center, Albuquerque, NM 87131, USA; 5 University of New Mexico-Kenya Programs, Kisumu and Siaya 40100, Kenya

**Keywords:** N95 respirator, decontamination, electret charge, filtration efficiency, quantitative fit test, strap mechanical properties

## Abstract

Shortages of N95 respirators for use by medical personnel have driven consideration of novel conservation strategies, including decontamination for reuse and extended use. Decontamination methods listed as promising by the Centers for Disease Control and Prevention (CDC) (vaporous hydrogen peroxide (VHP), wet heat, ultraviolet irradiation (UVI)) and several methods considered for low resource environments (bleach, isopropyl alcohol and detergent/soap) were studied for two commonly used surgical N95 respirators (3M™ 1860 and 1870+ Aura™). Although N95 filtration performance depends on the electrostatically charged electret filtration layer, the impact of decontamination on this layer is largely unexplored. As such, respirator performance following decontamination was assessed based on the fit, filtration efficiency, and pressure drop, along with the relationship between (1) surface charge of the electret layer, and (2) elastic properties of the straps. Decontamination with VHP, wet heat, UVI, and bleach did not degrade fit and filtration performance or electret charge. Isopropyl alcohol and soap significantly degraded fit, filtration performance, and electret charge. Pressure drop across the respirators was unchanged. Modest degradation of N95 strap elasticity was observed in mechanical fatigue testing, a model for repeated donnings and doffings. CDC recommended decontamination methods including VHP, wet heat, and UV light did not degrade N95 respirator fit or filtration performance in these tests. Extended use of N95 respirators may degrade strap elasticity, but a loss of face seal integrity should be apparent during user seal checks. NIOSH recommends performing user seal checks after every donning to detect loss of appropriate fit. Decontamination methods which degrade electret charge such as alcohols or detergents should not be used on N95 respirators. The loss of N95 performance due to electret degradation would not be apparent to a respirator user or evident during a negative pressure user seal check.

## Impact statement

Novel methods to extend limited supplies of respirators due to continuing shortages need more data to understand their impact on N95 performance. This article demonstrates two mechanisms for degradation: (1) loss of N95 strap elasticity due to extended use and (2) loss of electret charge causing severe degradation of filtration efficiency. While degraded strap performance would be apparent during a user seal check of a respirator, the loss of charge on the electret filtration layer did not change the pressure drop across the respirator and hence would not be apparent during a user seal check. Respirator users need to be aware of the potential hidden danger of exposure of an N95 respirator to alcohol, detergent, or other decontamination methods that disrupt the filtration layer charge. Vapor hydrogen peroxide, wet heat, UV, and bleach did not degrade N95 performance. These results can guide effective methods to conserve N95 supplies.

## Introduction

The 2020 global shortage of personal protective equipment (PPE) including filtering facepiece respirators (FFR) or N95 respirators has generated intense interest in methods to decontaminate respirators for reuse. Previous work by the Institute of Medicine predicted that a 42-day outbreak of a respiratory virus would require more than 90 million N95 respirators.^1^ Though FFRs are meant to be discarded after a single use, the Centers for Disease Control and Prevention (CDC) acknowledged that decontamination and reuse may need to be considered to ensure availability for health care personnel.^2^ Before utilizing a decontamination protocol, the CDC recommends that the method be evaluated for the ability to retain: (1) filtration performance, (2) fit characteristics, and (3) safety (inactivation of SARS-CoV-2).^1^

The Centers for Disease Control recommended three decontamination methods as promising: vaporous hydrogen peroxide (VHP), wet heat, and continuous ultraviolet radiation (UV, 254 nm).^2^ Other methods that have been proposed include the use of ethylene oxide (EtOH), autoclaving, bleach, liquid hydrogen peroxide, plasma hydrogen peroxide, isopropanol, ethanol, dry heat, and pulsed UV.^3–5^ Several methods including VHP, plasma hydrogen peroxide, and UV light have received Emergency Use Authorizations from the Food and Drug Administration (FDA) for decontamination of N95 respirators.^6–8^ Over the past decade, multiple studies have investigated the sterilization effectiveness for multiple decontamination methods using both viral surrogates,^7,9–11^ and more recently on SARS-CoV-2.^12–14^

However, the impact of different decontamination methods on the fit and structural integrity of N95 respirators is less well understood. The National Institute for Occupational Safety and Health (NIOSH) has provided a foundation for the filtration performance of decontaminated respirators with a wide range of decontamination methods.^3,15,16^ More recent work has also measured the fit of the respirator on the user, either with quantitative fit testing using a TSI PortaCount Respirator Fit Tester,^14,17^ or with qualitative fit testing using saccharin or bitrex.^5^ Some decontamination methods were found to degrade fit and/or filtration of respirators such as plasma hydrogen peroxide,^5,16^ dry heat^11,18^ and alcohol.^3,14^ Several decontamination methods, including vapor hydrogen peroxide, are widely seen to retain respirator fit and filtration performance.^4,13,14,15^ Vapor hydrogen peroxide was the decontamination method chosen for further study at the University of New Mexico Hospital^19^ and has been widely implemented by Battelle.^6^

An additional difficulty to understanding the effectiveness of decontamination methods is the strong dependence of potential adverse effects on the specific respirator manufacturer. Vapor hydrogen peroxide is not effective on respirators containing cellulose or activated carbon.^20^ Pleated respirator designs appear to be more resistant to high-heat decontamination than molded designs.^13^ Studies examining multiple respirator types found that degradation patterns were design specific.^16^ Additionally, very few published studies have examined the integrity of the elastic straps of the respirator,^21^ although for VHP, visual observations of elastic strap degradation after 30 decontamination cycles informed the upper limit on the number of potential decontamination cycles.^4^

A key element of the filtration efficacy of most N95 respirators is the use of an electrostatically charged melt-blown polypropylene filtration layer known as an electret.^22^ An electret is a permanently charged dielectric material. The electrostatic charge improves the filtration efficiency for submicron aerosol particles,^23^ allowing the respirator to achieve high filtration efficiency while maintaining a low pressure drop.^24^ The electrostatic charge layer improves filtration performance by both coulomb attraction and an induction effect where the filter charge induces a charge on the aerosol particle.^25^

The impact of decontamination methods on the electret filtration layer is not well understood, especially for N95 respirators. Studies on dust masks and other filters have shown that the electrostatic charge can be responsible for up to 69% of the filtration efficacy of filters that contain an electret.^23,25,26^ Studies on N95 respirators have postulated that loss of electrostatic charge was responsible for filtration degradation,^3,11^ but little-to-no data are available on measurements of the electric charge for the electret filtration layers in N95 respirators following decontamination.

As such, the impact of decontamination on N95 respirator performance was investigated with a specific focus on electret charge and mechanical strap properties. A range of decontamination methods (i.e. vapor hydrogen peroxide (VHP), wet heat, ultraviolet irradiation (UV), sodium hypochlorite (bleach), isopropyl alcohol (IPA), and detergent (soap) was studied on two models of N95 respirators (i.e. 3M™ 1860 and 1870+ Aura™). Performance was measured by quantitative fit testing, filtration efficiency testing, and measurement of the mechanical properties of the straps. The electrostatic potential of each respirator layer was measured and correlated with fit and filtration. At the commencement of this study, there was limited data available on decontamination of 3M™ 1870+ Aura™ Surgical N95 respirators, a commonly used FFR throughout many health care settings. Therefore, a major focus of this study was to collect performance data for that respirator model for multiple decontamination methods.

## Materials and methods

Because of the scarcity of PPE during the COVID-19 pandemic, there is a shortage of available N95 respirators for testing and ethical concerns about sacrificing new (un-used) respirators for our investigations. As such, initial testing of the impact of decontamination methods on electrostatic charge was performed using three highly charged filtration materials being considered for alternative face coverings. Results on the filtration materials guided the experimental design on a limited number of N95 respirators and are provided in the Supplementary Materials Section 1 (Figures S1–S4). Decontamination and performance testing were performed on two models of 3M™ respirator: 1860 and 1870+ Aura™. The 3M™ 1860 respirators utilize a molded cup design and have flat braided elastic straps approximately 1 × 5 mm in cross section. The 3M™ 1870+ Aura™ utilizes a flat folded design with flat rubber straps approximately 0.3 × 6 mm in cross section.

Respirators were collected at the University of New Mexico Hospital (UNMH) following decontamination using vapor hydrogen peroxide for one to two cycles as described in Perkins *et al.*^19^ Some of those respirators were then exposed to additional decontamination treatments as described below. In total, we received 3M™ 1870 (*n* = 11) and 3M™ 1860 (*n* = 7) which were supplemented with new 3M™ 1870+ Aura™ (*n* = 2) and additional new 3M™ 1860 (*n* = 5) respirators to complete the design of experiments.

### Filtration efficiency and pressure drop

The filter penetration testbed (FPT) was designed and built to characterize the aerosol performance of various material types for use on respirator filtration media (Figure S5). The system was equipped with instrumentation that resembled, but is not fully equivalent to, the FDA approved guidance in NIOSH procedure No. TEB-APR-STP-0059 (Code of Federal Regulation 42 CFR, Part 84, subpart K, §84.181) for N95 series respirators for informational purposes only.^27^ A list of the instrumentation used on the FPT system, in comparison to the NIOSH guidelines, is provided in the Table S1. The FPT system generates a 0.25% polydispersed sodium chloride mixture using a TSI Model 3076 Constant Output Atomizer as the challenge aerosol (distribution shown in Figure S6). The aerosol was charge neutralized to a Boltzmann equilibrium state using a Haug ionizer as mentioned in the NIOSH guidelines. Because this system uses a smaller respirator sample (47 mm) than the NIOSH guidelines (102 mm), the flow rate was modified to achieve an equivalent filter face velocity of 17.3 cm/s. Pressure drop across the respirator sample was measured using a Dwyer Magnehelic® differential pressure gauge (Model 2010). The pressure-drop measurement was important to understand performance effects of average inhalation and exhalation resistance, 343.2 Pa and 245.1 Pa, respectively.^28^ Finally, efficiency was calculated from the ratio of upstream and downstream particle concentrations of the aerosol using a TSI Model 3022 A condensation particle counter (CPC).

### Respirator quantitative fit testing

Quantitative Fit Testing was performed using a PortaCount® Pro+ Respirator Fit Tester Model 8038. The PortaCount® is an ambient aerosol condensation nuclei counter that samples the particle concentration inside an N95 respirator and in the environment to calculate a quantitative fit factor. Respirators were tested using static headforms as recommended by the National Personal Protective Technology Laboratory (NPPTL) for decontaminated N95 respirators.^29^ Several static headforms were created to support the study. The most successful commercial headform was an injection training headform (Mediarchitect UL079 Injection Training Mannequin Face Model, Life Size) which had a soft rubber “skin” coating over rigid skeleton features. Headforms were also 3D printed using anthropometric data provided by National Institute for Occupational Safety and Health.^30^ The challenge was to mimic the texture of human skin, which has a Shore Hardness of around 30.^31^ The most successful 3D printed implementation with the most compliant face used Agilus 30 rubber as a 4 mm thick skin over an inner core of Stratsys PolyJet SUP706 Soluble Support Material. Images of respirators fitted on representative headforms are shown in Figure S7.

Each manikin had a breathing tube that connected to the mouth as well as a sampling tube for the PortaCount® lateral left of the nose. Breathing was replicated using a Harvard Apparatus Dual Phase Control Respirator. The breath volume was set at a tidal volume of 750 mL at 15 breaths per minute to replicate the normal breathing flow conditions of 11.2 liters per minute. Following the NPPTL protocol, the N95 respirator was placed on the head form and adjusted using the training feature in the Fit Pro+ software (v3.3).^32^ Three attempts were made to adjust the nose bridge and straps to achieve a passing score. The Manikin Fit Factor (MFF) was measured using the TSI Fit Pro “Fast Test protocol” ^32^ which averages respirator particle counts over 2 min of “normal” breathing. Data presented here for the 3M™ 1870+ Aura™ respirators were collected on the injection training headform. None of the headforms were able to achieve passing fit scores on the 3M™ 1860/1860S respirators.

### Electrostatic surface charge

Electrostatic testing was performed using two electrostatic voltmeters: (1) Trek 344 non-contact voltmeter, which has a ≈ 1 cm cylindrical probe located ∼2 mm from the testing surface, and a maximum potential of 2000 V, and (2) Trek 821HH contact voltmeter with a thin (<1 mm) cylindrical probe placed on the measurement surface which measures up to 2430 V. Both probes operate by adjusting the potential of the probe to match the surface potential, nullifying the electric field between the two surfaces.^33^ Both voltmeters were connected to the building earth ground and probes were grounded prior to measurements.

Electrostatic potential measurements were performed on 47 mm disks taken from the respirators. The respirator layers were separated and placed on a grounded surface for at least 30 min to equilibrate to room conditions. Measurements were performed under controlled temperature (20–21°C) and relative humidity (range: 45–51%). Each layer was held between three insulated alligator clips. Potential measurements were made using both voltmeters in the center and three locations in between the attachment points on both sides of each layer and averaged.

Surface potential measurements were highly variable, but this is not unexpected. Prior single fiber charge measurements on electrets found that the degree and polarity of charge can vary along an individual fiber and among adjacent fibers resulting in fluctuations in the measured macroscopic electric potential.^34^ Prior macroscopic measurements on electrets using a non-contact voltmeter found measurement standard deviations of ±300–500 V.^35^

### Mechanical properties

Mechanical measurements of the respirator straps were performed on an Anton Paar Modular Compact Rheometer (MCR702) fitted with a TwinDrive linear stage. A piece of elastic strap 40 mm long was excised and mounted in the rheometer tensile grips. Extensional storage and loss moduli of the straps were measured in oscillation over a range of amplitudes and frequencies. The average storage and loss moduli over the range of 0.1–1.0% strain were compared for the different decontamination conditions. Stress relaxation was measured by extending the straps to 10% deformation and calculating the fraction of the stress that relaxed after a 2-min hold.

Fatigue testing was performed on similar respirator strap samples. To estimate the test parameters that simulate respirator donnings, the extension of the respirator straps placed on a static headform was measured. The upper strap of a 3M™ 1870+ Aura™ was extended to 2.3× its original length when the respirator fitted on the headform. The lower strap was extended only 1.5× its original length when placed on the neck but would also have to be stretched to 2.5× the original length to pass over the top of the head during donning of the respirator. To simulate repeated donnings with mechanical testing, the strap sample was stretched to 150% strain (2.5× the original length) and held for 10 min and then brought back to 0% strain and allowed to relax for 10 min. The stretch cycle was repeated for 10 cycles and then the strap length and linear mechanical properties were remeasured before continuing fatigue testing.

### Decontamination methods

Decontamination methods were selected to investigate the CDC’s recommended procedures,^2^ options for low-resource settings, and processes believed to degrade surface charge on the electret filtration layer.

### Vaporous hydrogen peroxide

Respirators were decontaminated using a BioQuell Clarus C system at University of New Mexico Hospital as described in detail by Perkins *et al.*.^19^ The process consisted of conditioning, (10 min), pre-gassing, gassing (83 minutes), gassing dwell (36 min), and aeration to remove residual hydrogen peroxide. VHP is one of the decontamination methods labeled as most promising by the CDC.^2^

### Wet heat

Respirators were placed in a humidity-controlled chamber (Memmert HCP 150) at 60°C and 85% relative humidity. The oven required ∼5–10 minutes to recover to a relative humidity of 75% after loading the samples. The respirators were placed in the chamber for ∼40 min and then placed in a fume hood to dry. Wet heat is one of the decontamination methods labeled as most promising by the CDC.^2^

### Ultraviolet irradiation (UVI or UV)

Commercial equipment is available for UV sterilization as described by other researchers.^3,16^ A proposed low-resource method to sterilize respirators is to use the UV lamp in standard biosafety cabinets for a treatment of 15 min per side. Respirators were UV treated in a Thermo Forma Class II A/B3 biological safety cabinet with integrated UV lamp (new bulb installed one month before processing) for sterilization at 254 nm. The UV light intensity was measured to be 116 µW/cm^2^ using a Thor Labs PM1000 with a 200–1000 nm sensor. The UV dose was calculated to be 0.2 J/cm^2^ which is significantly lower than commercial UV sterilizers and prior studies.^3,21^ UV is one of the decontamination methods labeled as most promising by the CDC^2^ and is also an option for low-resource environments.

### Sodium hypochlorite (*bleach)*

Respirators were soaked in a 10 wt% solution of Clorox Germicidal Bleach in DI water (0.6 wt% NaOCl) for 30 min. The bleach solution did not completely wet the respirator since the respirators remained buoyant (presumably due to trapped air). The respirators were weighed down with a smaller glass beaker containing some bleach solution. Because of concerns with residual chlorine on bleach-soaked respirators and potential off-gassing for the user, the use of a dilute hydrogen peroxide rinse was explored which is discussed in the Supplementary Material. Bleach has been shown to effectively inactivate a wide range of pathogens on N95 respirators ^9,11^ though concerns have been raised about damage to the respirator.^16,36^ If effective, bleach decontamination of respirators would be helpful in low-resource environments.

### Isopropyl alcohol

Respirators were soaked in isopropyl alcohol for 30 min (LabChem ACS grade). The IPA completely wet the respirator and it did not need to be weighed down in the liquid. Respirators were drained and allowed to dry overnight in a fume hood. Isopropyl alcohol has been found to degrade electrostatic charge held on electret filter fibers^25,37^ and was found to partially dissolve the safety notices printed on the respirators.

### Detergent (soap)

Respirators were soaked in a 2 wt% solution of Dawn dish detergent in DI water for 30 min. The mask was then drained and rinsed in a container of fresh DI water and allowed to soak for 20 min. Though the aqueous solution soaked completely into the respirator, it was still secured in place with a small beaker to ensure complete immersion. Detergent solutions have been found to degrade N95 filtration performance with a hypothesized cause of electret charge degradation.^3^ Soap has been shown to reduce filtration efficiency of FFRs.^3^ In coupon studies, discussed in section 1 of the Supplementary Material, detergent was the most effective at neutralizing electret charge.

## Results

The performance testing of decontaminated N95 respirators sought to answer three questions:

1. Are 3M™ 1870+ Aura respirators robust to several decontamination methods?
2. Does electret charge correlate with changes in filtration efficiency in N95 respirators?
3. How do the elastic straps respond to decontamination?

The two respirator models differed in their construction. The 3M™ 1870+ Aura consisted of four layers though one stiff structural layer was only found in the central panel of the folding respirator. The filter is polypropylene covered with polypropylene coverweb^38^ (no composition was listed for the structural layer). The straps are polyisoprene fastened with steel staples.^38^ The 3M™ 1860 contained three layers with the polypropylene filtration layer sandwiched between a polypropylene coverweb and a polyester shell.^39^ The straps are made of braided polyisoprene fastened by steel staples.^39^ Digital microscope images (Figure S8) and fiber size measurements (Table S2) of all the layers are provided in section 3 of the Supplementary Material. Both respirators contain the same 3M™ proprietary filtration layer known as the Advanced Electrostatic Media which uses a high level of electrostatic charge to enhance capture efficiency of airborne particles.^24^

All decontaminated 3M™ 1870+ Aura respirators received one to two treatments of VHP in addition to one treatment of the alternate methods. For the 3M™ 1860 respirators, the wet heat and bleach respirators also received one to two VHP treatments. The controls and 3M™ 1860 respirators treated with UV, IPA and Soap were not used and did not receive any VHP treatments. A summary of respirators used and their decontamination history is provided in Table S5.

### Filtration efficiency, pressure drop, and quantitative fit testing

**Figure 1.**
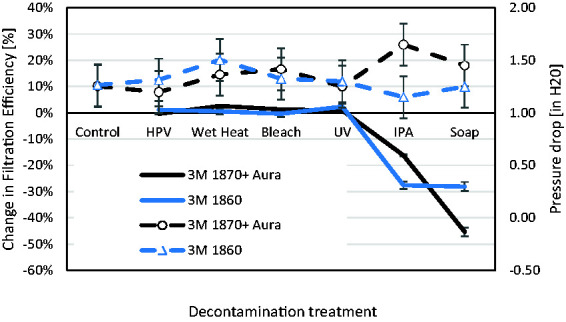
Change in filtration efficiency (solid line) and pressure drop (dashed line) as a function of decontamination treatment for 3M™ 1870+ Aura™ and 1860 respirators. Error bars of change in efficiency are the standard deviation. Error bars on the pressure drop are the measurement uncertainty reported by the pressure gauge manufacturer. (A color version of this figure is available in the online journal.)

Results from filtration efficiency and pressure drop testing of both models of respirators are summarized in Figure 1. The CDC recommended decontamination methods including vaporous hydrogen peroxide (VHP), wet heat, UV and bleach had no impact on filtration efficacy, or pressure drop for the 3M™ 1870+ Aura and 3M™ 1860 N95 respirators. No change in respirator performance was observed after VHP decontamination, enabling the use of VHP-treated respirators to study other decontamination treatments.

Accurate quantitative fit testing could only be achieved for the 3M™ 1870+ Aura respirators. Results of the Manikin Fit Factor testing are shown in Table 1. Respirators exposed to vaporous hydrogen peroxide (VHP), wet heat, UV, and bleach all maintained their filtration efficiency and passed fit testing with the majority hitting the maximum Manikin Fit Factor (MFF) measurement of 200+.

**Table 1.** Summary of quantitative fit test results for manakin fit factor of 3M™ 1870+ Aura respirators.

3M™ 1870 (# respirators)	Manikin fit factor
Control (2)	200+
VHP (4)	200+
Wet Heat + VHP (2)	150/200+
Bleach + VHP (2)	200+
UV + VHP (1)	200+
IPA + VHP (1)	39
Soap + VHP (1)	10

The isopropanol (IPA) and soap treatments allow the exploration of the relationship between the electrostatic charge and filtration efficiency in N95 respirators. As observed previously,^3^ the filtration efficiency dropped significantly for both cases. The respirators exposed to IPA and soap also no longer passed the quantitative fit test. Note that the loss of filtration function was not associated with significant changes in the pressure drop across the filtration media. Thus, the loss of N95 performance would not be apparent to a respirator user or evident during a negative pressure user seal check.

### Electret surface charge measurement

**Figure 2.**
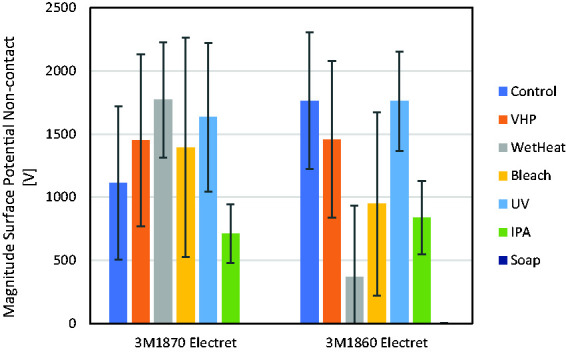
Magnitude of the surface potential measured with the Trek 344 non-contact voltmeter for multiple decontamination methods. Upper measurement limit was 2000 V. Soap-treated respirators had electret surface potentials of 0 ± 0 V for 3M™ 1870+ Aura and 1 ± 1 V for 3M™ 1860 which is plotted but not visible on the graph. Error bars show the standard deviation.

The overall results for the change in electrostatic charge magnitude of the electret filtration layer after decontamination are shown in Figure 2. Testing results for electrostatic charge on all layers for both respirators with contact and non-contact voltmeters are included in Section 4 of the Supplementary Materials. As seen previously reported,^35^ variability in these readings results in standard deviations over 500 V in many cases, which is believed to be primarily caused by variation in the charge imparted to the electret.^34^ It was not uncommon to approach the upper limit of the surface potential reading for the noncontact voltmeter which may bias the variability lower in those cases.

**Figure 3.**
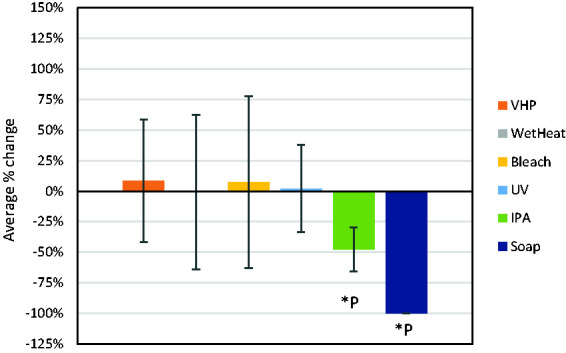
Average percent change in magnitude of surface potential for electret filtration layer for the 3M™ 1870+ Aura™ and 1860 N95 respirators after various decontamination treatments. Error bars show standard deviation calculated by propagation of errors. *P denotes a statistically significant change from the control.

Since the two models are known to contain the same filtration layer,^24^ results for the two respirators are averaged in Figure 3 to reduce fluctuations due to the small number of samples and inherent variability in the measurement. The only decontamination methods with statistically significant changes in electret charge were isopropanol and soap (as denoted with a *P in the figure).

The isopropanol and soap treatments allow exploration of the relationship between the electrostatic charge and filtration efficiency in N95 respirators. As observed previously, the filtration efficiency dropped significantly for both cases. The electrostatic charge measurements showed that the isopropanol reduced the surface charge by ≈ 50% and the soap completely neutralized the surface potential of the electret filtration layer. These data show that the loss of filtration efficiency observed in the previous section was correlated with a decrease in electret charge for these respirators. These data support the hypothesis of Viscusi *et al.*^3^ that electret degradation is the cause for N95 performance degradation in N95 respirators. Changes in filtration efficiency and electret charge imply that the electrostatic charge was responsible for 28% and 45% of the overall filtration efficiency for the 3M™ 1860 and 1870+ Aura™, respectively.

### Elastic strap mechanical measurement

**Figure 4.**
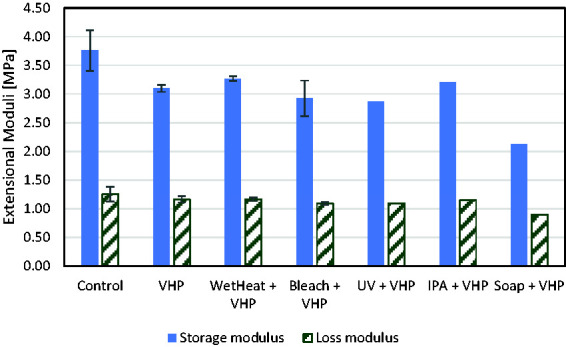
Extensional moduli for the 3M™ 1870+ Aura™ N95 respirator after decontamination. (A color version of this figure is available in the online journal.)

**Figure 5.**
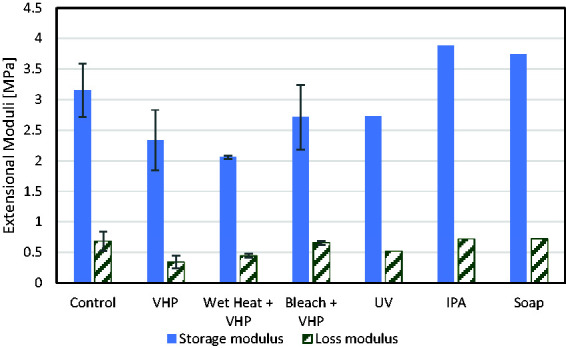
Extensional moduli for the 3M™ 1860 N95 respirator straps after decontamination. Note UV, IPA, and soap tests were performed on new respirators that had not been previously used or received VHP decontamination. (A color version of this figure is available in the online journal.)

Mechanical measurements of extensional storage and loss moduli are shown in Figures 4 and 5 for the 3M™ 1870+ Aura™ and 3M™ 1860 respirators, respectively. Measurements were taken of one strap sample from each respirator except for the controls where two samples from each respirator were tested. Error bars show the standard deviation where multiple respirators were tested under the same condition. Both respirator straps behave as viscoelastic solids because the storage and loss moduli are of similar magnitude with the storage contribution dominating. The 3M™ 1870+ Aura™ respirator strap has a larger dissipation (loss) fraction and had more stress relaxation when held extended compared to the 3M™ 1860.

With the limited numbers of samples, it is difficult to quantify statistical significance of the differences observed since two samples do not provide a complete sampling of the distribution. To improve statistical comparisons, control respirators were each tested twice, but the variability between respirators is much larger than the variability between tests on the same respirator. Half of the decontaminated 3M™ 1870+ Aura™ respirators straps had a lower storage modulus than the controls by a statistically significant amount with a two-tailed *t*-test as described in Section 5 of the Supplementary Material. Most of the 3M™ 1860 respirator straps did not show significant change in properties after decontamination.

**Figure 6.**
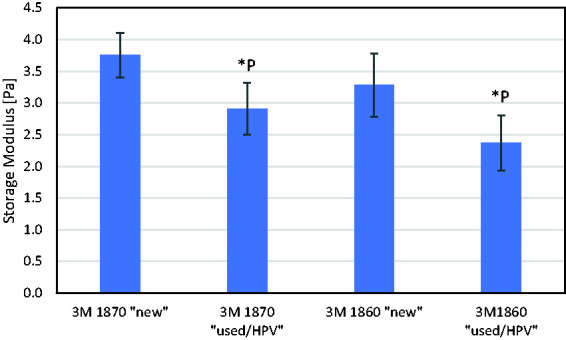
Storage modulus for N95 respirator elastic straps groups into “new” and “used/HPV” cohorts. Error bars show the standard deviation. *P denotes a statistically significant change from the “new” cohort. (A color version of this figure is available in the online journal.)

By combining samples into larger groupings, clearer trends can be observed. One grouping of interest is between used/post-hospital respirators and “new” respirators which have only received a small number of donnings during quantitative fit tests. All the used/post-hospital respirators had received VHP decontamination. “New” respirators included controls and 3M™ 1860 respirators used for UV, IPA, and soap treatments. The mean of the two groupings, as well as the probability that the difference in the means is due to chance (*P*-value) are shown in Figure 6 and Table S4. In this comparison, the used respirators had a statistically significant decrease in storage modulus relative to the “new” respirators. It is not clear if the cause of the degradation was the repeated use of the respirators in the hospital environment or the decontamination. The decrease in mechanical properties was different than the small increase in stress measured after repeated VHP decontamination.^40^ The decrease was not sufficient to affect quantitative fit test scores but it is possible that mechanical damage could accumulate. Repeated donnings of respirators and vapor hydrogen peroxide decontamination have both been noted to cause damage to the elastic straps which may limit the number of potential wear/decontamination cycles of N95 respirators.^4,41,42^

To separate the effects of multiple donnings and VHP decontamination, we performed additional tests and analysis. Separating the groups of used/VHP respirators into groups that had received one VHP cycle and a group that had received two VHP cycles found no statistically significant difference in mechanical properties between the two groups. To simulate the process of repeated donnings, samples of a control of each model of respirator were measured after repeated cycles of stretching to 150% strain. This strain was chosen after measurements of how stretched a strap became during donning onto a headform during fit testing. It is also one of the two strain values recommended by the National Personal Protective Equipment Laboratory for certification of decontaminated N95 respirators.^29^

**Figure 7.**
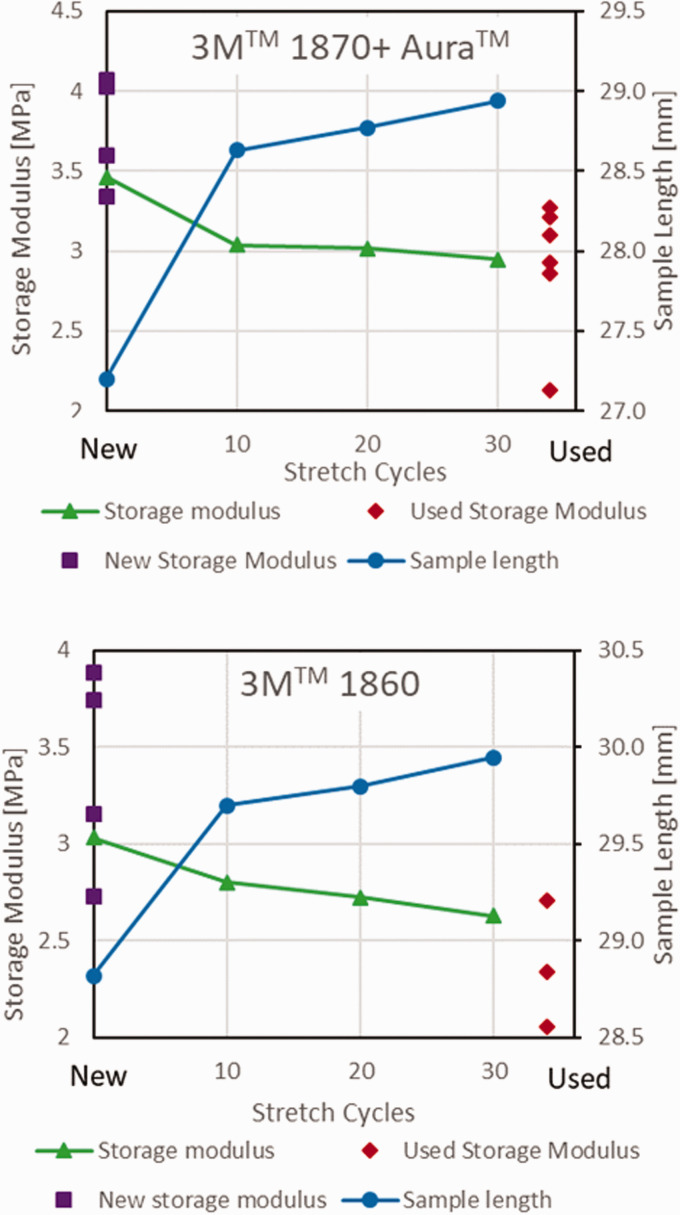
The storage modulus and sample length as a function of repeated stretching to 150% strain for 3M™ 1870+ Aura™ and 1860 respirator straps. The storage moduli of the “used/VHP” respirators from the decontamination study are plotted with diamonds for reference and the “New” (control) respirators are plotted as squares. (A color version of this figure is available in the online journal.)

Figure 7 shows the evolution of the storage modulus and sample length as a function of the number of cycles. After repeated stretching, the strap experiences stretching that does not recover in time (plastic deformation) resulting in increased length over cycles. The storage modulus also decreases with increasing number of cycles. The change in strap cross section was not accounted for in the storage modulus measurement to avoid handling and remounting the strap repeatedly. The 6% increase in length would be expected to cause a roughly 6% decrease in cross sectional area which could account for half of the observed decrease in effective storage modulus. For reference, the storage moduli of the “used/VHP” cohort are also plotted on the right demonstrating that the degradation in modulus observed after 30 stretch cycles is of a similar magnitude as the decrease observed in the “used” cohort.

## Discussion

Results of testing decontamination methods for N95 respirators showed that the filtration efficiency, pressure drop, and quantitative fit were robust for several methods including vapor hydrogen peroxide, wet heat, bleach, and ultraviolet light. Response to multiple decontamination methods is provided for the 3M™ 1870+ Aura™ FFR which previously had limited published data available. While UV did not result in degradation, the UV intensity of a biosafety cabinet may not have been of sufficient intensity to provide sterilization for an N95 respirator.

The degradation of electrostatic charge of the electret filtration layer was correlated with a decrease in the quantitative fit test and filtration efficiency of the respirator. The two treatments chosen to study electret charge, isopropyl alcohol and soap solution, both showed significant degradation of quantitative fit test, filtration efficiency, and electrostatic charge of the filtration later. Electrostatic filtration was responsible for approximately 1/3 of the filtration efficiency for the N95 respirators tested. It is important to note that the loss of filtration function after electret degradation was not associated with significant changes in the pressure drop, and as such, loss of performance would not be apparent to a respirator user or evident during a negative pressure user seal check.

Degradation was observed in the mechanical properties with a small degree of softening of the extensional storage modulus. Although the observed changes were not enough to affect respirator fit, continued degradation could be problematic for respirator fit. Fatigue measurements of the strap material and subsequent statistical analysis indicated that the degradation was due to repeated stretching of the straps. Thus, the extended use or reuse of the N95 respirator may be more damaging than many decontamination processes. Loss of fit due to decrease of strap elasticity should be apparent to a respirator user during a negative pressure user seal check.

Significant variation between respirators under the same decontamination conditions was observed in both mechanical and electrostatic charge measurements. General variability of the electrostatic charge measurements was also a challenge for determining statistically significant trends in the results. Stronger evidence for these observations could be gathered by testing larger cohorts of N95 respirators and developing more robust electrostatic measurement methods.

CDC recommended decontamination methods including VHP, wet heat, and UV light did not degrade N95 respirator fit or filtration performance in these tests. Extended use of N95 respirators may degrade strap elasticity, but a loss of face seal integrity should be apparent during user seal checks. NIOSH recommends performing user seal checks after every donning^43^ to detect loss of appropriate fit. Decontamination methods which degrade electret charge such as alcohols or detergents should not be used on N95 respirators.

## Supplemental Material

sj-pdf-1-ebm-10.1177_1535370220976386 - Supplemental material for COVID-19 global pandemic planning: Performance and electret charge of N95 respirators after recommended decontamination methods Click here for additional data file. Supplemental material, sj-pdf-1-ebm-10.1177_1535370220976386 for COVID-19 global pandemic planning: Performance and electret charge of N95 respirators after recommended decontamination methods by Anne M Grillet, Martin B Nemer, Steven Storch, Andres L Sanchez, Edward S Piekos, Jonathan Leonard, Ivy Hurwitz and Douglas J Perkins in Experimental Biology and Medicine
