# Supplementary material for: COVID-19 global pandemic planning: Performance and electret charge of N95 respirators after recommended decontamination methods
Source: Exp Biol Med (Maywood). 2020 Dec 16;246(6):740–8. doi: 10.1177/1535370220976386 (PMC7961645; doi:10.1177/1535370220976386)
Supplement: sj-pdf-1-ebm-10.1177_1535370220976386 - Supplemental material for COVID-19 global pandemic planning: Performance and electret charge of N95 respirators after recommended decontamination methods [file sj-pdf-1-ebm-10.1177_1535370220976386.pdf]

Supplementary Information for COVID-19 Global Pandemic Planning: Performance and Electret Charge of N95 Respirators after Recommended Decontamination Methods

Anne M. Grillet, Martin B. Nemer, Steven Storch, Andres L. Sanchez, Edward S. Piekos, Jonathan Leonard, Ivy Hurwitz & Douglas J. Perkins

1. Initial Screening of Decontamination Methods with Coupons:

Coupon studies were utilized to explore a wider range of potential decontamination methods for their potential impact on the electret layer prior to testing the limited supply of N95 respirators. Three electrostatically active materials were tested: NP097 filtration fabric, Swiffer dry cleaning wipes and a polyester filter fabric. While it is unclear whether such materials are true electrets, these materials were thought to be effective for screening the impact of decontamination treatments on electrostatically charged nonwoven fibers.

NP097 (NanoNxt, distributed by Filti) filtration material is advertised as a filtration element for face covering, and also is being considered for 3D printed respirators. It consists of a polypropylene outer layer, nanofiber center layer and a polyester inner layer. The manufacturer claims a 95% filtration efficiency, though those results are not guaranteed to be representative of all Filti material [1].

Swiffer dry cleaning wipes (Procter & Gamble) are of unknown composition though the manufacturer advertises that the charge properties help improve dust collection.

The polyester filter fabric is sold by McMaster Carr under part number 92255T71. It is 100% polyester and is advertised to remove particles down to 50 microns.

The materials were exposed to the following decontamination methods: wet heat, UV, bleach, isopropyl alcohol and soap solution, as described in the manuscript. Measurement of all three of the materials following decontamination revealed a significant initial impact on the electrostatic charge (Figure S1). In addition, the following decontamination methods were also tested on the coupons:

Dry Heat: Coupons were placed in a laboratory oven (Fisher Scientific IsoTemp oven Model 6916) at 75°C for 30 minutes and then placed in a fume hood to dry.

Ethanol (EtOH): Coupons were submerged in a solution of 70wt% ethanol (Sigma Aldrich Supelco liquid chromatography grade) solution in DI water for 30 minutes and then placed in a fume hood to dry overnight.

Liquid Hydrogen Peroxide: Coupons were submerged in a 6wt% hydrogen peroxide solution in DI water for 30 minutes and then placed in a fume hood to dry overnight. Solutions were mixed immediately prior to use. Coupons remained buoyant and had to be held submerged with tweezers.

Bleach + H_2O_2 Rinse: Coupons were soaked in a 10wt% solution of Clorox Germicidal Bleach in DI water (0.6wt% NaOCl) for 30 minutes. To address concerns about residual chlorine after bleach treatment, the coupons were subsequently soaked in a 0.3wt% hydrogen peroxide solution in DI water for 30 minutes. The bleach and hydrogen peroxide solutions did not completely wet the coupons. Since the coupons were buoyant (presumable due to trapped air) they were weighed down with tweezers.

Electrostatic measurements were made as described in the manuscript. The square coupons were mounted using insulated clips at each corner and 5 measurements were made per side (center and middle of each side of the square). Results for the magnitude of the surface charge, as well as the average change in the surface charge after treatment are shown in Figure S1 Figure S3. All three materials exhibited an initial surface charge of ≈ 750V. The first three treatments shown are isopropyl alcohol (IPA), 70% ethanol (EtOH) and soap. All were expected to degrade the electrostatic charge based on previous reports [2-4]. As observed for the respirators, the soap completely neutralized the surface charge. The isopropanol degraded the surface charge, but not to the extent previously reported [3, 5, 6]. Conversely, ethanol did not degrade the electric charge. For the Swiffer Dry, the electric charge increased substantially after ethanol exposure. Figure S4 shows the correlation between individual measurements made on the Swiffer Dry control samples to demonstrate the significant correlation between the two electrostatic voltmeters, despite the difference in magnitude observed.

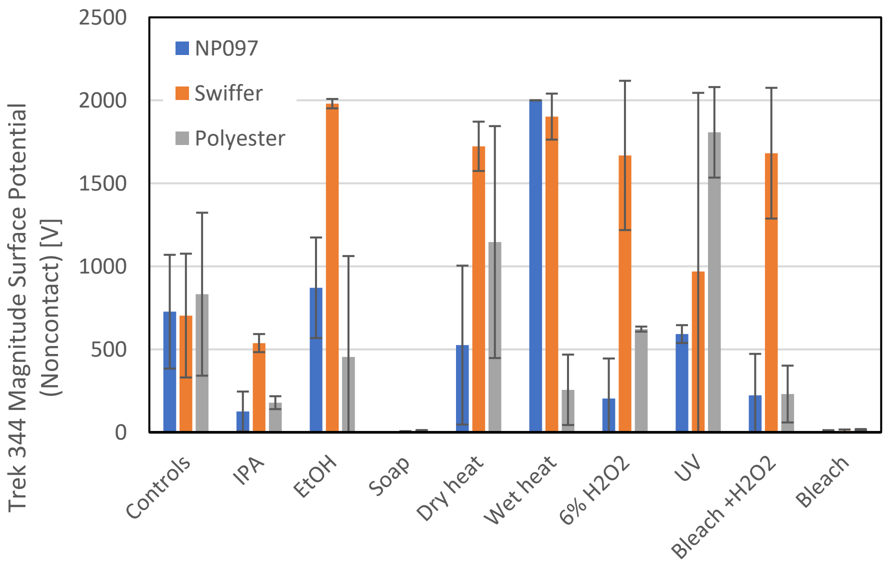

Figure S1: Magnitude of the surface charge as a function of treatment for three charged materials measured with non-contact electrostatic voltmeter. Error bars are the standard deviation of the measurements.

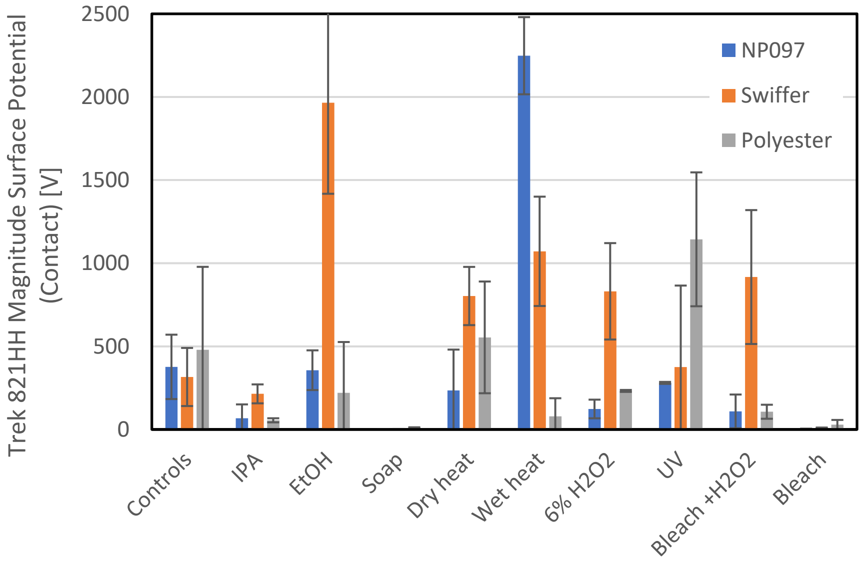

Figure S2: Magnitude of the surface charge as a function of treatment for three charged materials measured with contact electrostatic voltmeter. Error bars are the standard deviation of the measurements.

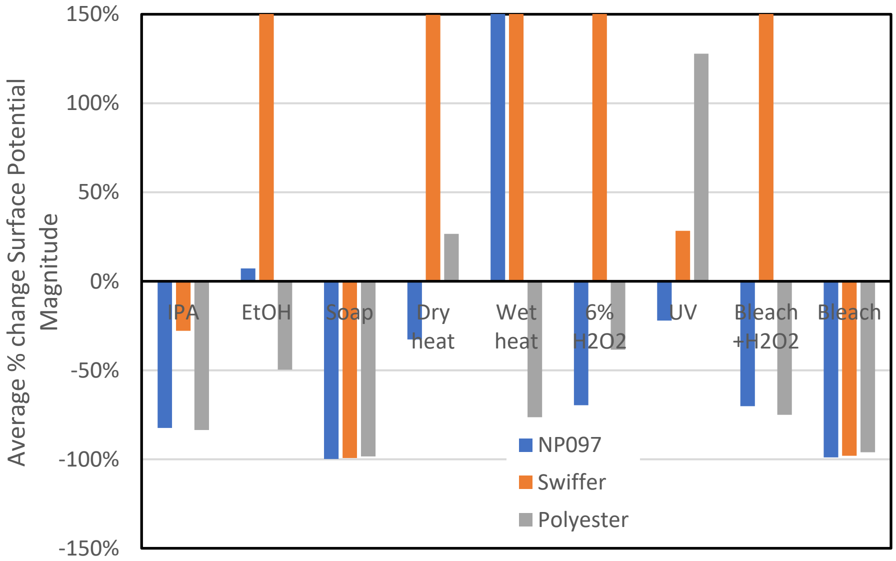

Figure S3: Average % change in the magnitude of the surface charge as a function of treatments applied to three charged materials. Results are the average of both contact and non-contact measurements.

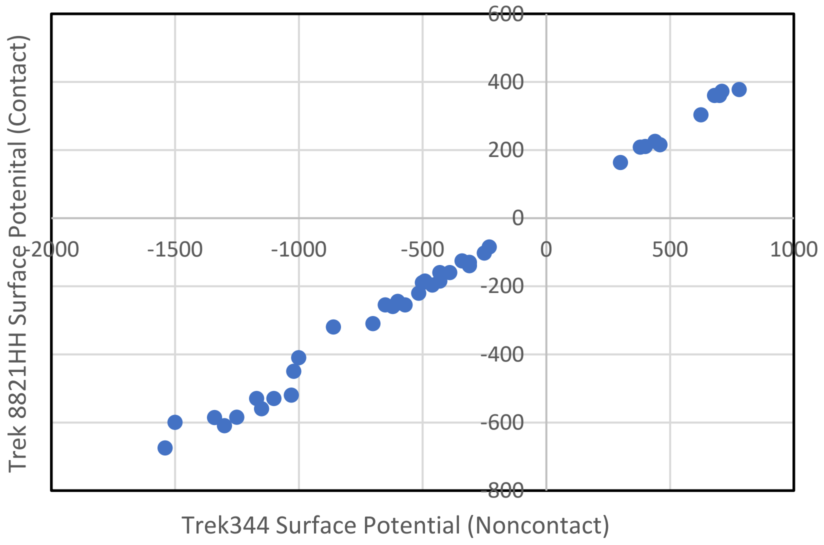

Figure S4: Correlation between surface potentials measured with contact and non-contact electrostatic voltmeters. 40 individual measurements taken on 4 control samples of Swiffer Dry.

For the decontamination treatments, the changes in electrostatic charge results were mixed. Dry heat and UV light were either neutral or beneficial. Wet heat and liquid hydrogen peroxide were either beneficial or detrimental depending on the coupon material. The results of the bleach treatment were not as expected. Bleach alone dramatically neutralized the electric surface charge on all three materials. When identical bleach treatments were followed by a rinse with dilute hydrogen peroxide, the degradation was not as severe, and the charge actually increased in the case of the Swiffer Dry. In intact respirators, previous studies showed that bleach decontamination had no effect [7, 8] or caused only a small decrease in filtration performance after repeated treatments [9].

We chose the decontamination methods for the study of N95 respirators based on the results described herein. In addition to the three methods recommended by the Centers for Disease Control and Prevention [10], tests were also performed on three additional treatments: bleach was tested to explore the electret charge in a respirator exposed to bleach solution; and isopropanol and soap solutions were chosen to specifically degrade electret charge and explore the relationship with filtration efficiency.

2. Filtration Efficiency Testing

The Filter Penetration Testbed (FPT) is shown in Figure S5. Filtration efficiencies are reported as the average of at least 3 measurements for each respirator sample. The pressure drop was measured once for each respirator sample. Error bars shown are taken from the measurement accuracy of the Dwyer Model 2010 Magnehelic® differential pressure gauge (±2% of 10 inches of water or 0.2 inches H_20 pressure). NIOSH guidelines recommend testing at a mean number averaged particle size of 75nm which is overlapped by this particle size distribution. The mean number average particle in the FPT is 40nm so results cannot be quantitatively compared to the NIOSH testing criteria. As an option, the system was built to generate a monodisperse aerosol using the TSI Model 3080 Electrostatic Classifier and TSI Model 8081L Differential Mobility Analyzer, where the particles are selected to determine penetration efficiency at different sizes, however, this was not used in this study.

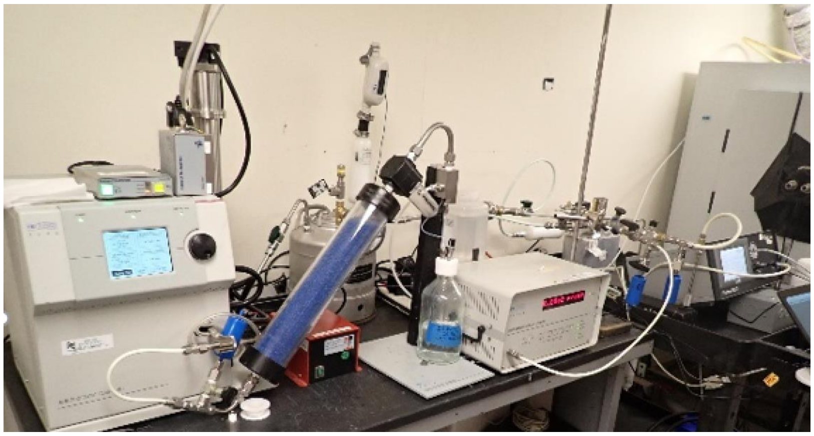

Figure S5: Filter Penetration Testbed (FPT)

Table S1 shows the components of the Filter Penetration Testbed and also provides a more detailed comparison between the capabilities of this system and the NIOSH guidance for testing of N95 respirators (Code of Federal Regulation 42 CFR, Part 84, subpart K, §84.181 [11]). The average particle size distribution used in filtration efficiency testing is shown in Figure S6.

Table S1: Filter Penetration Testbed components and capabilities compared with NIOSH guidelines for testing of N95 respirators. For testing, the Omega pressure transducers were replaced with the Dwyer Model 2010 Magnehelic® differential pressure gauge.

Row	Measurement	NIOSH Criteria (42 CFR, Part 84, Subpart K, §84.181)	FPT Comparison Discription
1	**Airflow**	1. +/-2% Accuracy<br>2. Challenge Air Flow: 85LPM (Filter Face Velocity: 17.337 cm/s)<br>3. Particle Loading Air Flow: 30 LPM (Filter Face Velocity: 6.119 cm/s)	Air flow controlled by Alicat Scientific Model MCR at recommended filter face velocities, while flow is monitored by a TSI Model 4040.
2	**Pressure**	1. +/-2% Accuracy<br>2. Flow Resistance: 25 mmHg (Inhalation) and 35mmHg (Exhalation)	Pressure monitored by Omega Model PX-409 Pressure Transducer and confirmed with a magnehelic pressure gauge.
3	**Aerosol Generation**	2% NaCl solution in DI H2O. Particle Size Distribution (PSD) with a median count diameter of 75 nm, Standard Deviation of 1.86. Aerosol should be neutralized to Boltzmann equilibrium state	Aerosol generated using a TSI Model 3076 Constant Output Atomizer and size selected using a TSI Model 3080 Electrostatic Classifier with a TSI Model 8081L Differential Mobility Analyzer (DMA), as needed. Aerosol neutralized to Boltzmann equilibrium state using a Haug Neutralizer.
4	**Data Acquisition**	Thermal printer or optional Data Acquisition System (DAQ)	No specific data acquisition system used.
5	**Particle Sizing & Counting**	Penetration can be measured to 0.001%, Efficiency to 99.999%, TSI Model 8130 or 8130A-Automated Filter Tester	Particulates are sized in two ways with a (1) TSI Scanning Mobility Particle Sizer (SMPS, 3010) and a Palas Promo 2000-Aerosol Spectrometer System. For the penetration study, particle counting utilizes a TSI Model 3022A Condensation Particle Counter (CPC), which measures concentration upstream and down stream of the filter housing.
6	**Microbalance**	Accurate to 0.0001 grams (g)	Filter pre and post-weights were taken with a Mettler Toledo AX205 (Max Capacity 220g, d=0.01mg)
7	**Concentration**	Aerosol should not exceed 200mg/m^3	Concentration well below max concentration, which were confirmed by weight.
8	**Particle Loading**	200 mg +/- 5 mg, at a flow of 30LPM and 40 minutes	Particle loading was not taken into consideration, since the exposure concentrations were much lowered that the NIOSH criteria. Also, if size selection was conducted, this further decreased the overall exposure concentration.
9	**Filter Dimensions**	102 mm circular	Filter housing used was 47 mm. Flow was converted to filter face velocity to account for surface area difference.
10	**Control Filter Material**	Pall Type A/E glass fiber filter, high efficiency with 1 micron pore size	Pall Type A/E glass fiber filter (P/N 61631) used as a control.
11	**Climate Parameters**	Testbed to be maintained at a RH of 30 ±10% and temperature of 25 ±5 °C	Temperature and relative humidity monitored by Omega Temp/RH probe that was inline with FPT air flow. (Range: 2 to 98% RH and -17 to 49°C)
		Samples preconditioned at a Relative Humidity (RH) of 85 ±5%and temperature of 38 ±2.5 °C	MicroClimate Temperature/Humidity Chamber (MC-3): Temperature -68C to 190C.<br>RH 0-100% RH.

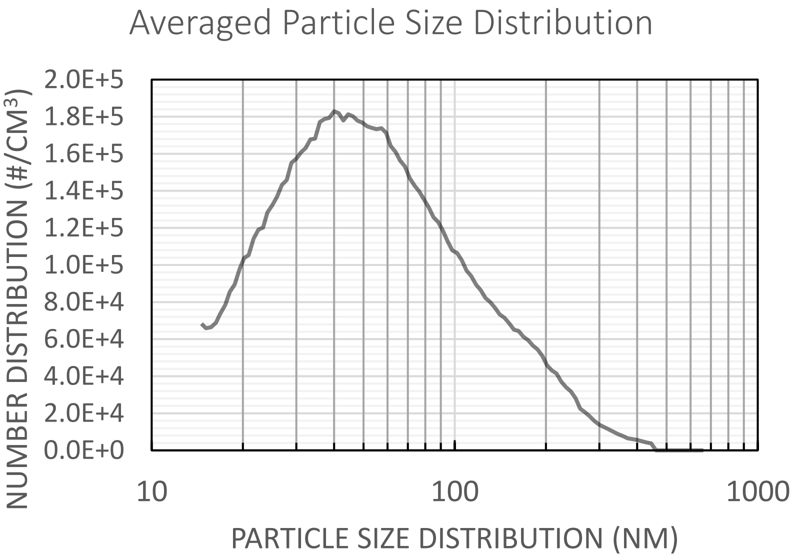

Figure S6: Average particle size distribution used in filtration efficiency studies. NIOSH guidelines recommend testing at 75-300nm which is overlapped by this particle size distribution though results cannot be quantitatively compared to the NIOSH standard because the peak of the distribution is 40-50nm instead of 75nm.

3. Composition of N95 respirators

The two respirators used in the manuscript are shown in Figure S7. The 3M^{TM} 1870 has a design that folds flat with flaps that open to conform to the face. The 3M^{TM} 1860 has a rigid cup design. Both have a moldable nose bridge and foam along the inside of the mask at the nose.

Imaging of each of the 3M^{TM} respirator models was performed on a Keyence VHX6000 digital microscope using a 250-2500x high magnification lens and reflected light source. Images shown in Figure S8 were captured using the Keyence composite three dimensional imaging and high dynamic range (HDR) features to collapse the low density non-woven fiber fabric to a single focal plane and enhance clarity of individual fibers. Measurements of average fiber diameter are shown in Table S2.

3M^{TM} 1870+ Aura N95 surgical respirator **3M^{TM} 1860 N95 surgical respirator**

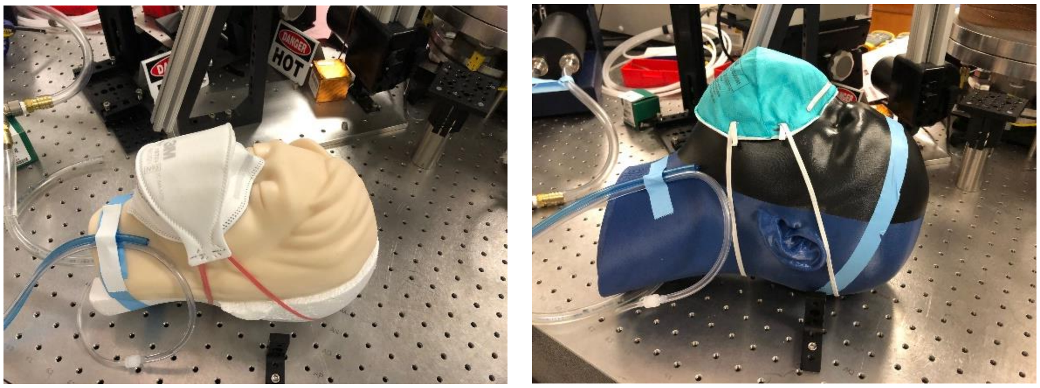

Figure S7: N95 surgical respirators used in decontamination study displayed on manikin head forms used for quantitative fit testing.

3M™ 1870+ Aura N95 surgical respirator | **3M™ 1860 N95 surgical respirator**

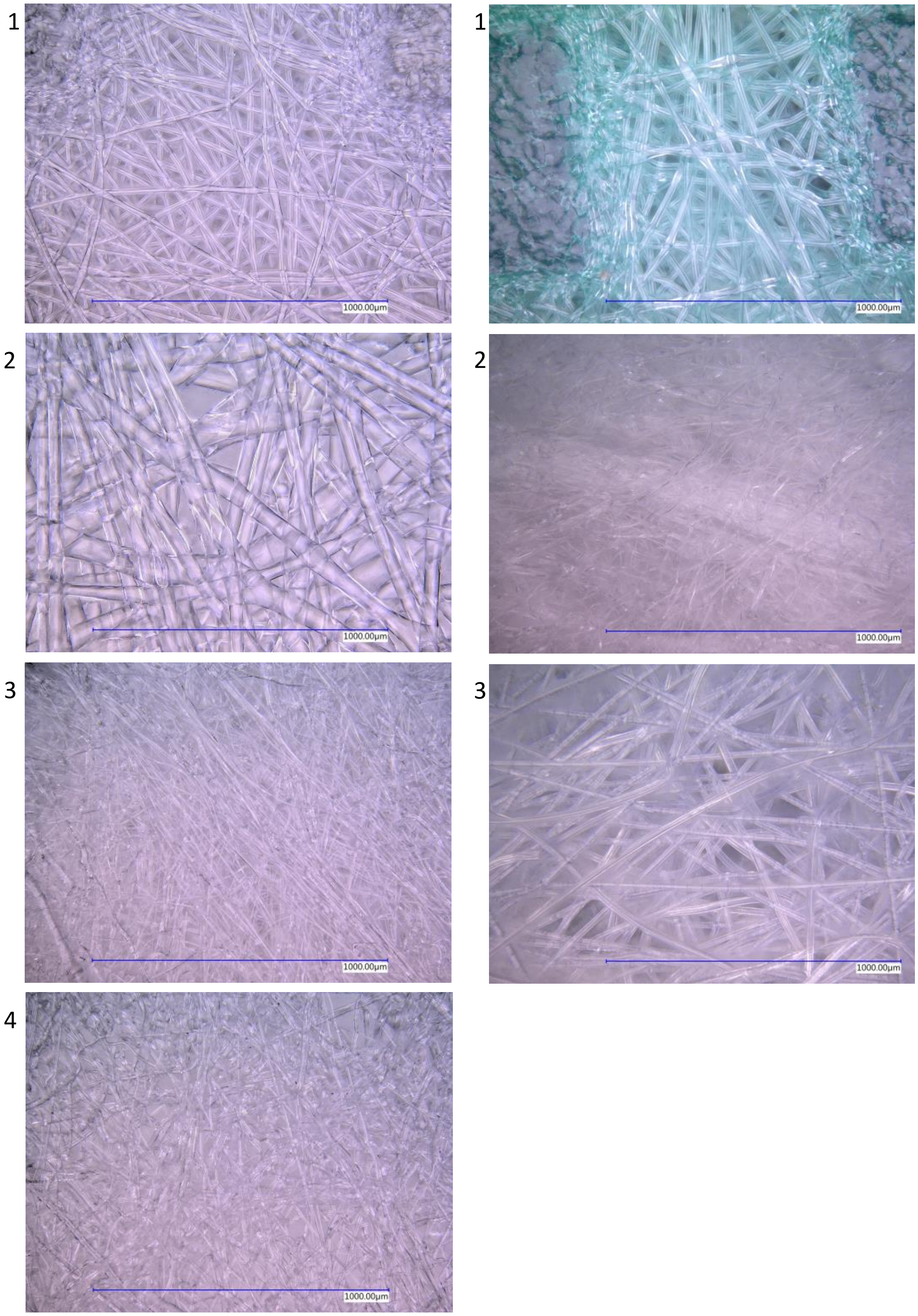

Figure S8: Digital microscopy images of the layers found in the 3M™ 1860 and 1870+ Aura™ surgical respirators.

The 3M™ 1870+ Aura™ consists of 4 primary layers. Layer one on the outside of the respirator is a low density non-woven polypropylene coverweb [12] with adhesion points where the fibers are melted

together to form a physical crosslink as seen on the two sides of the first row image in Figure S8. Layer two is only found in the center portion of the mask and is a thin rigid layer with a single layer of large fibers presumably to provide structural support. Layer 3 is a dense non-woven fabric of fine fibers identified as the polypropylene Advanced Electrostatic Media filtration layer [12, 13]. This layer can be separated into sublayers, but they all appear to be identical in morphology and thus were not separated for this study. This layer is fragile, and fibers can be easily dislodged. Layer 4 is a paper-thin layer and may play a role in protecting the electret layer. The low fiber density and open pores of layers 1 and 2 suggest they have a limited role in aerosol filtration performance.

The 3M™ 1860 consists of three main layers. Layer one is a low-density nonwoven fiber polypropylene coverweb [14] with many adhesion points that act as physical cross links. These cross links are believed to provide mechanical stability. Layer 2 is a dense non-woven fabric of fine fibers identified as the polypropylene Advanced Electrostatic Media filtration layer [13, 14]. This layer can be separated into sublayers, but they all appear to be identical in morphology. This layer is fragile, and fibers can separate easily. Layer 3 was a stiff nonwoven polyester fiber shell [14] and provides the support for the cup shaped design of the respirator. The large open pores of Layers 1 & 3 suggest they have a limited role in aerosol filtration performance. Average fiber sizes are shown for both N95 models in Table S2 and a summary of respirators used in decontamination testing are shown in Table S3.

Table S2: Average fiber size for each layer of N95 respirator.

Layer	3M™ 1870+ Aura™	3M™ 1860
1	16.6±1.5 micron	19.9±0.9 micron
2	51.6±7.9 micron	2.6±1.2 micron
3	4.6±2.9 micron	20.6±2.3 micron
4	6.8±3.7 micron	

Table S3:Summary of N95 respirators used for each decontamination method. For previously used N95s, the number of additional HPV cycles they received is shown in parentheses.

	# N95s (# HPV cycles)	
	3M™ 1870+ Aura™	3M™ 1860
Control	2 (0)	2 (0)
HPV	2 (1 & 2)	2 (1 & 2)
Wet heat	2 (1 & 2)	2 (2 & 2)
Bleach	2 (1 & 2)	2 (1 & 2)
UV	1 (1)	1 (0)
IPA	1 (2)	1 (0)
Soap	1 (2)	1 (0)

4. Study of Decontamination on N95 Respirator Electrostatic Charge

Electrostatic charge measurements were made on all the layers of each respirator, in addition to the measurements on the electret filtration layer presented in the manuscript. Here we provide the results of both the Trek 344 non-contact voltmeter and the Trek 821HH voltmeter separately, as well as the average change in the measured magnitude of electrostatic charge relative to the controls for each layer as a function the decontamination technique.

The electrostatic surface charge of the 3M™ 1870+ Aura™ respirator layers measured with the non-contact and contact voltmeters are shown in Figure S9 and Figure S10, respectively. All layers showed electrostatic surface charges and, as previously discussed, the charges were quite variable with significant standard deviations and many measurements reaching the maximum potential of the voltmeters. Figure S11 displays the mean percentage change averaged over the two voltmeter readings and statistically significant changes are marked with *P.

Similar data for the 3M™ 1860 respirator is presented in Figure S12-Figure S14. The innermost polyester layer did not have an appreciable surface charge.

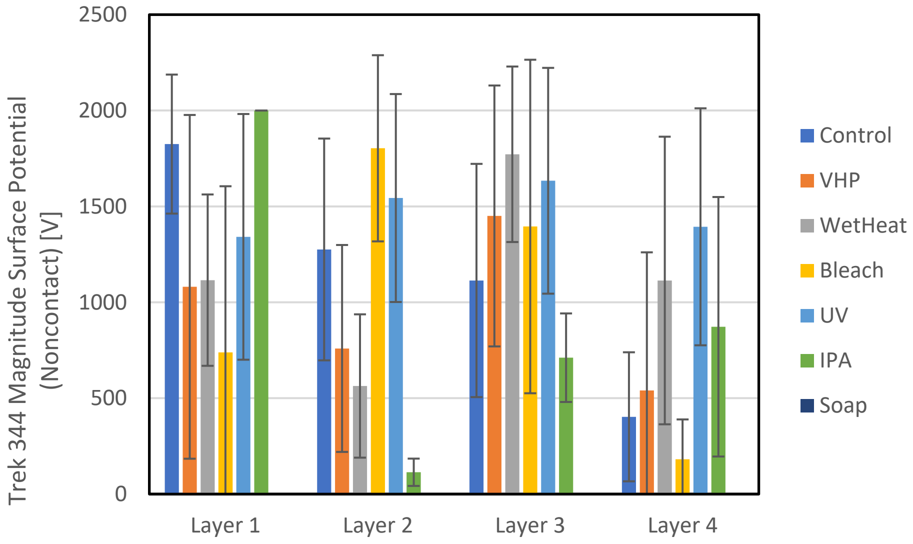

Figure S9: Magnitude of the surface potential for 3M™ 1870+ Aura™ respirator layers from the Trek 344 non-contact electrostatic voltmeter. Maximum reading of 2000 V was reached frequently. Layer 1 is the outermost and Layer 4 is the innermost. Layer 3 is the electret filtration layer. Error bars show the standard deviation of the measured potentials.

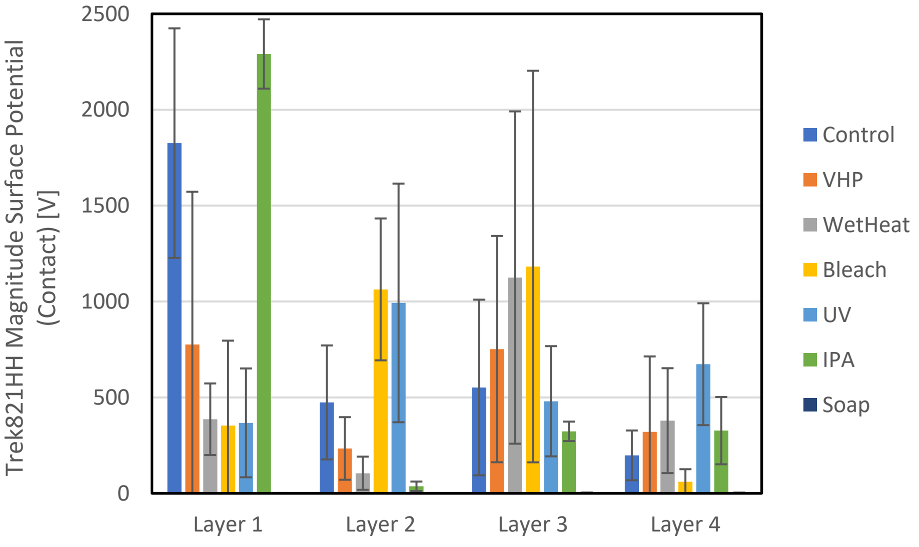

Figure S10: Magnitude of the surface potential for 3M™ 1870+ Aura™ respirator layers from the Trek 821HH contact electrostatic voltmeter. Maximum reading of 2430 V was reached on Layer 1. Layer 1 is the outermost and Layer 4 is the innermost. Layer 3 is the electret filtration layer. Error bars show the standard deviation of the measured potentials.

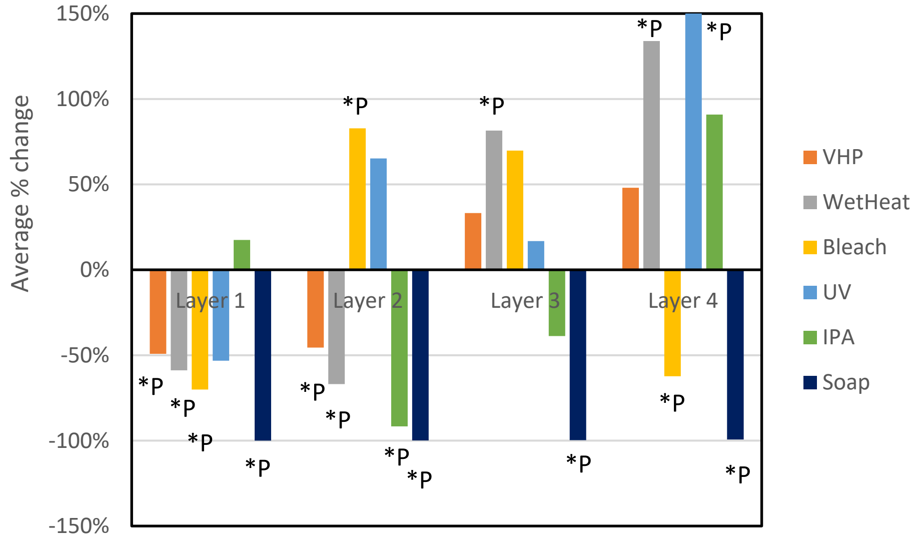

*Figure S11: Average percentage change in the magnitude of the surface potential for 3M™ 1870+ Aura™ respirator layers after various decontamination treatments. *P indicates a statistically significant difference between the mean of the decontaminated material and control for both voltmeters.*

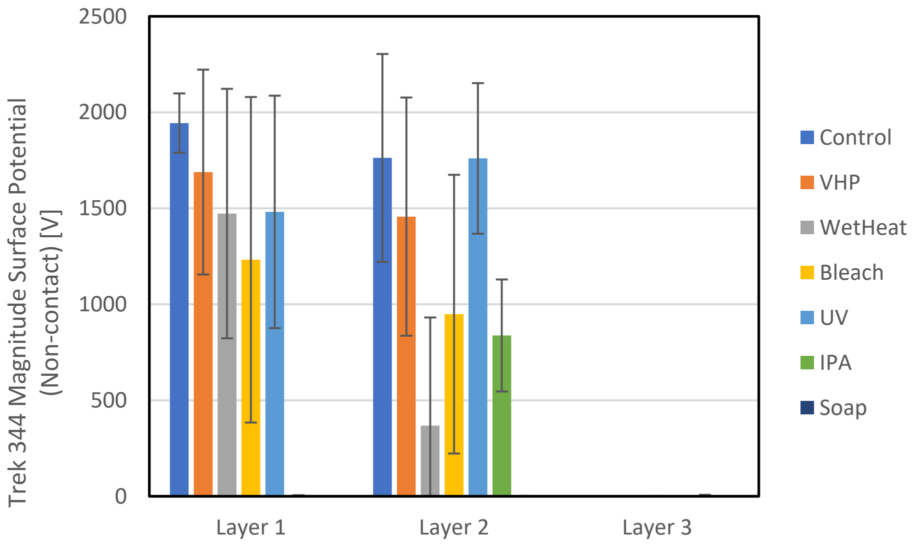

Figure S12: Magnitude of the surface potential for 3M^{TM} 1860 respirator layers from the Trek 344 non-contact electrostatic voltmeter. Maximum reading of 2000 V was reached frequently. Layer 1 is the outermost and Layer 3 is the innermost. Layer 2 is the electret filtration layer. Error bars show the standard deviation of the measured potentials.

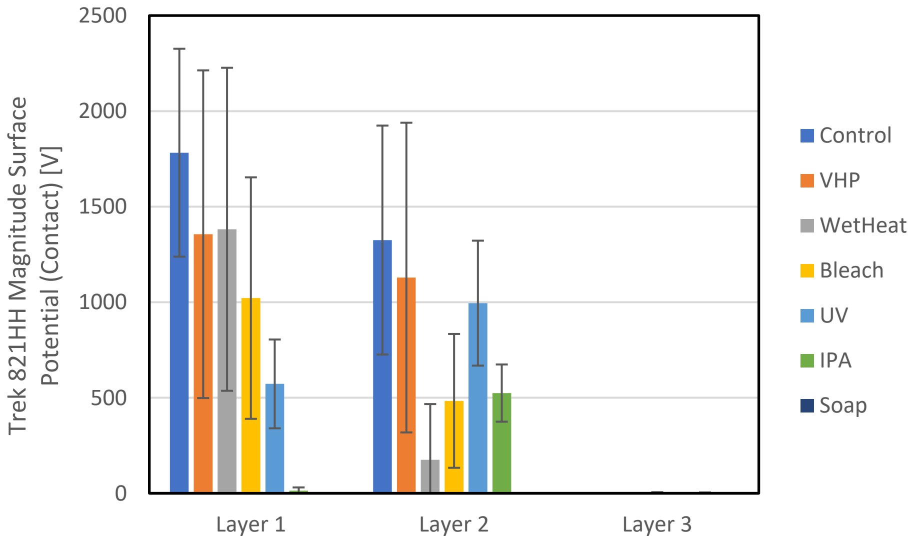

Figure S13: Magnitude of the surface potential for 3M^{TM} 1860 respirator layers from the Trek 821HH contact electrostatic voltmeter. Maximum reading of 2430 V was reached frequently for Layer 1 and a few times for Layer 2. Layer 1 is the outermost and Layer 3 is the innermost. Layer 2 is the electret filtration layer. Error bars show the standard deviation of the measured potentials.

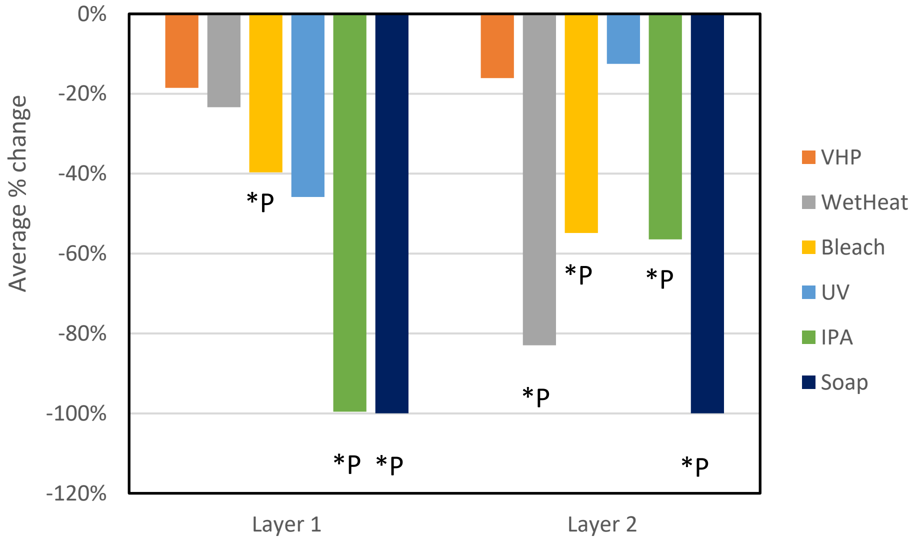

*Figure S14: Average percentage change in the magnitude of the surface potential of 3M™ 1860 respirator layers 1 & 2 after various decontamination treatments. *P indicates a statistically significant difference between the mean of the decontaminated material and control for both voltmeters.*

Table S4: Summary of mechanical properties for "new" and "used / VHP" cohorts of N95 respirators. A comparison with a p-value of less than 0.05 was deemed statistically significant (marked in blue in table).

Respirator / property	"New"	"Used / VHP"	p-value
3M™ 1870+ # FFRs	2 (4 tests)	10	
Storage Modulus	3.76±0.35 MPa	2.91±0.25 MPa	0.007
Loss Modulus	1.25±0.12 MPa	1.11±0.05 MPa	0.117
Stress relaxation	8.8±0.6%	10.2±0.7 %	0.018
3M™ 1860 # FFRs	5 (7 tests)	6	
Storage Modulus	3.28±0.37 MPa	2.37±0.35 MPa	0.006
Loss Modulus	0.67±0.10 MPa	0.48±0.12 MPa	0.041
Stress relaxation	5.4±0.4%	5.9±0.2 %	0.056

5. Statistical Significance Testing Process:

Statistical comparison of two means was determined by assuming the variance of the control and decontamination populations could be different. For two populations A (control samples) and B (decontaminated samples), the T value was defined as:

$$T = \frac{\mu_B - \mu_A}{\sqrt{\frac{\sigma_A^2}{N_A} + \frac{\sigma_B^2}{N_B}}}$$

where μ_x is the mean of population x, σ_x is the standard deviation and N_x is the number of samples in group x [15]. The effective degrees of freedom df can be calculated using:

$$\mathrm{df} = \frac{\left(\frac{\sigma_A^2}{N_A}+\frac{\sigma_B^2}{N_B}\right)^2}{\frac{\left(\frac{\sigma_A^2}{N_A}\right)^2}{N_A-1}+\frac{\left(\frac{\sigma_B^2}{N_B}\right)^2}{N_B-1}},$$

which is the formula used in Microsoft Excel. The probability that the difference in the two populations was due to chance p-value was then calculated using the Excel two-tailed T distribution function:

p-value = T.DIST.2T(ABS(T),df)

Probabilities of random chance below 5% were classified as statistically significant. For the electrostatic measurements, both the noncontact and contact voltmeters needed to register statistically significant changes for the overall result to be classified as statistically significant.

Propagation of error [15] was used to calculate the standard deviation of averages and percent change of measured quantities using the following formulas:

Average f(x,y) = (x+y)/2 : $\sigma_f = \frac{1}{2}\sqrt{\sigma_x^2 + \sigma_y^2}$

Percent change f(x,y) = 100*(x-y)/y : $\sigma_f = 100 * \sqrt{\frac{\sigma_x^2}{y^2} + \frac{x^2\sigma_y^2}{y^4}}$

References:

1. *Filti Face Mask Material Data Sheet*. 2020.
2. Viscusi, D.J., William P. King, and R.E. Shaffer, *Effect of Decontamination on the Filtration Efficiency of Two Filtering Facepiece Respirator Models.* Journal of the International Society for Respiratory Protection, 2007. **24**: p. 93.
3. Chen, C.-C. and S.-H. Huang, *The Effects of Particle Charge on the Performance of a Filtering Facepiece.* American Industrial Hygiene Association Journal, 1998. **59**(4): p. 227-233.
4. Kim, J., W. Jasper, and J. Hinestroza, *Direct probing of solvent-induced charge degradation in polypropylene electret fibres via electrostatic force microscopy.* Journal of Microscopy, 2007. **225**(1): p. 72-79.
5. Chen , C.C., M. Lehtimäki, and K. Willeke, *Loading and Filtration Characteristics of Filtering Facepieces.* American Industrial Hygiene Association Journal, 1993. **54**(2).
6. Sanchez, A.L., et al., *Experimental Study of Electrostatic Aerosol Filtration at Moderate Filter Face Velocity.* Aerosol Science and Technology, 2013. **47**: p. 606-615.
7. Vo, E., S. Rengasamy, and R. Shaffer, *Development of a Test System To Evaluate Procedures for Decontamination of Respirators Containing Viral Droplets.* Applied and Environmental Microbiology, 2009. **75**(23): p. 7303-7309.
8. Viscusi, D.J., et al., *Evaluation of Five Decontamination Methods for Filtering Facepiece Respirators* Ann. Occup. Hyg, 2009. **53**(8): p. 815–827.
9. Bergman, M.S., et al., *Evaluation of Multiple (3-Cycle) Decontamination Processing for Filtering Facepiece Respirators.* Journal of Engineered Fibers and Fabrics 2010. **5**(4).
10. Centers for Disease Control and Prevention. *Decontamination and Reuse of Filtering Facepiece Respirators*. 2020 [cited 2020 April 3, 2020]; Available from: https://www.cdc.gov/coronavirus/2019-ncov/hcp/ppe-strategy/decontamination-reuse-respirators.html.
11. *Electronic Code of Federal Regulations: Part 84—Approval of Respiratory Protective Devices*. 2019 December 13, 2019 March 27, 2020]; Available from: https://www.ecfr.gov/cgi-bin/text-idx?SID=3fa5fa13db7aad46a8456116fae8a43e&m=12&d=13&y=2019&pd=20200520&pitd=20200520&node=pt42.1.84&submit=GO#se42.1.84_1181
12. *Technical Specification Sheet 3M™ Aura™ Health Care Particulate Respirator and Surgical Mask, 1870+, N95.* 2017.
13. *3M™ Health Care Particulate Respirator and Surgical Masks*. 2014.
14. *Technical Specificatoin Sheet for 3M™ Health Care Particular Respirator and Surgical Mask, 1860, N95.* 2017.
15. Box, G.E.P., W.G. Hunter, and J.S. Hunter, *Statistics for Experimenters: An introduction to Design, Data Analysis, and Model Building*. WIley Series in Probability and Mathematical Statistics. 1978, New York, NY: Wiley & Sons.

Sandia National Laboratories is a multimission laboratory managed and operated by National Technology & Engineering Solutions of Sandia, LLC, a wholly owned subsidiary of Honeywell International Inc., for the U.S. Department of Energy's National Nuclear Security Administration under contract DE-NA0003525.

This paper describes objective technical results and analysis. Any subjective views or opinions that might be expressed in the paper do not necessarily represent the views of the U.S. Department of Energy or the United States Government.
